# Self-Improvement for Team-Players: The Effects of Individual Effort on Aggregated Group Information

**DOI:** 10.1371/journal.pone.0011705

**Published:** 2010-07-21

**Authors:** Sean A. Rands

**Affiliations:** School of Veterinary Science, Centre for Behavioural Biology, University of Bristol, Bristol, United Kingdom; University of Utah, United States of America

## Abstract

By putting effort into behaviours like foraging or scanning for predators, an animal can improve the correctness of its personal information about the environment. For animals living in groups, the individual can gain further information if it is able to assess public information about the environment from other group members. Earlier work has shown that consensus group decisions based upon the public information available within the group are more likely to be correct than decisions based upon personal information alone, given that each individual in a group has a fixed probability of being correct. This study develops a model where group members are able to improve their personal likelihood of making a correct decision by conducting some level of (costly) effort. I demonstrate that there is an evolutionarily stable level of effort for all the individuals within the group, and the effort made by an individual should decrease with increasing group size. The relevance of these results to social decision making is discussed: in particular, these results are similar to standard theoretical predictions about the amount of vigilance shown by individuals decreasing with increasing group size. However, this model suggests that these results could come about where individuals are coordinating their effort within the group (unlike standard models, which assume that all individual effort is independent of the actions of others). This ties in with experimental findings where individuals have been shown to monitor the efforts of others.

## Introduction

All animals rely on being able to process information about the environment to make accurate decisions about their activities, such as where to find food, and what actions to take to avoid predators [1]. The accuracy of the individual's actions depends in part on the quality of the information it possesses, and therefore the animal should devote a significant amount of effort into sampling the environment: this improvement in ‘personal information’ should in turn lead to a reduction in the animal's chances of making an incorrect decision [2]. For animals living in groups, individuals can benefit from the information about the environment available from other group-members, which may be communicated between group members, or may simply be inadvertently available as ‘public information’ [1], [3].

The availability of public information and its uses in enhancing an individual's knowledge of its environment is therefore a potential benefit to the individual of associating in a group [4]. Usually, it is assumed that the group's members are always able to assess their environment to a set degree of certainty, and therefore each individual's personal information about the environment is just as likely to be correct as that of other group members. Following the jury theorem proposed by the Marquis de Condorcet [5], previous models have used this assumption to demonstrate how individual group-members could pool their personal information, and, by following the majority decision of the group, how each individual could increase its own chances of making a correct decision [6]–[8]. These models assume that the individuals in a group are able to signal some form of information about the environment (such as whether food is present or absent in a particular location, or a choice between two possible foraging sites). If each individual has a given probability of being correct (and is more than 50% likely to be correct), an individual following the decision shown by the majority of the group is more likely to be right than if it were to rely on just its own personal information.

However, individuals don't just receive information about their environment passively: an individual can increase the accuracy of its personal information by putting in some degree of effort in sampling the environment. If individuals within a group are able to increase their own certainty of making the correct decision, this in turn will mean that the majority decision of the group is more likely to be correct. Where there is some sort of cost involved with improving personal information, the individual therefore faces a trade-off: improving personal information is costly, but the resulting benefits could be received by both the individual and its fellow group-members. Because group-members are able to benefit from the actions of each other, there is potential for individuals to cheat, and free-load off the effort of others. Here, I demonstrate that the evolutionarily stable amount of effort that an individual should put into improving its own certainty of making the correct decision is directly linked to both the social and ecological constraints experienced by the individual, by considering the effects of group size, as well as the costs and benefits associated with improving information accuracy.

## Methods

The Condorcet model assumes that each individual possesses and signals a personal intention about an action relating to the environment, which has a given probability of being correct. In this case, the intention signalled is the individual's preferred choice out of a mutually exclusive pair of actions (where one of the actions is taken to be ‘correct’, and the other ‘incorrect’, *e.g.* deciding to visit a patch which may or may not be empty, or being vigilant or non-vigilant at a given level of predation risk). We assume that an individual is able to put some effort *e* (where *e*≥0) into reducing its uncertainty in the information it possesses about which of the pair of choices is correct: given that there is some basal accuracy *b* for an individual if it puts in no effort into improving its information (where 0≤*b*≤1), then we can write the individual's personal accuracy for putting effort *e* into improving the accuracy of the information (such as through sampling the environment) as
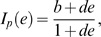
(1)where *d* is a scaling constant denoting the accuracy of a decision in relation to the effort (where *d*>0 – it is assumed here that the increase in accuracy will diminish as effort increases). Therefore, the probability of the information being correct falls between *b* and 1 depending upon the effort put in by the individual.

Given a population where all individuals put in effort *e_p_*, the likelihood of success when following the majority decision can be calculated [7] using the binomial distribution for a group consisting of *n* individuals:
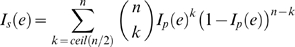
(2)where *ceil*(*n*) denotes that smallest possible integer value equal to or greater than *n* (necessary when *n* is even to avoid ‘hung’ votes where half of the group prefer each of the two options available).

If we assume that there is a single mutant in the group of size *n* that puts in effort *e_m_*, then it follows that if the mutant makes the correct decision, the majority decision will be correct if at least (*n*−1)/2 of the other members of the group also make the correct decision, which will occur with probability 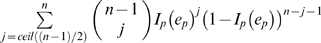. Similarly, if the mutant makes the wrong decision, the majority decision will still be correct provided that at least (*n*+1)/2 of the other members of the group are correct, which will occur with probability 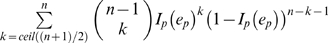. If we denote the probability of the mutant being correct as *I_p_*(*e_m_*), given that it puts effort *e_m_* into improving its own accuracy, then it follows that the group's overall likelihood of success is
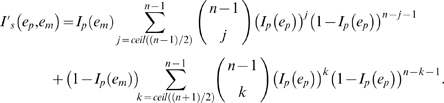
(3)We assume that there is a fitness cost *c*(*e*) to each individual of putting in effort *e* such that *c*(*e*) = *κe* (where the cost scalar *κ* is a constant), and a fitness gain from being correct such that *g*(*I_s_*) = *γI_s_* (where the gain scalar *γ* is a constant). We also assume that there are no costs associated with assessing the choice of action of the other members of the group (so public information is essentially free, which could occur if it was available as an inadvertent cue). Assuming that other members of the group are not related to the mutant, we can write the fitness of an mutant playing effort *e_m_* within a group where all other individuals play the population strategy *e_p_* as
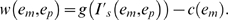
(4)Following standard procedures for calculating an evolutionarily stable strategy (ESS – [9]), a population ESS e* exists when 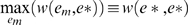. The best mutant effort *e_m_* to a given population effort *e_p_* is found by calculating *e_m_* where ∂*w*(*e_m_*,*e_p_*)/∂*e_m_* = 0 (holding *e_p_* constant, and confirming ∂^2^ *w*(*e_m_*,*e_p_*)/∂*e_m_* ^2^<0 and (∂^2^ *w*(*e_m_*,*e_p_*)/∂*e_m_*∂*e_p_* + ∂^2^ *w*(*e_m_*,*e_p_*)/∂*e_m_* ^2^)<0 to satisfy conditions for mutant and population stability [10]), and solving the resulting equation for *e_m_* = *e_p_* = e*. It can be demonstrated that the equations can be solved to give multiple solutions for e* across the real numbers, but we are only interested in those values of e* where e*>0, *w*(e*,e*)>0, and *I_p_*(e*)>0.5 (the accuracy of an individual playing strategy e* has to be greater than 0.5 for there to be any benefit to paying attention to a group consensus decision). Valid solutions for multiple parameter sets were calculated numerically – in all cases, a maximum of one existing value of e* could be found when these constraints were applied. The effects of changing each variable individually (keeping the other parameters constant) were investigated in 5000 parameter sets.

## Results

**Figure 1:**
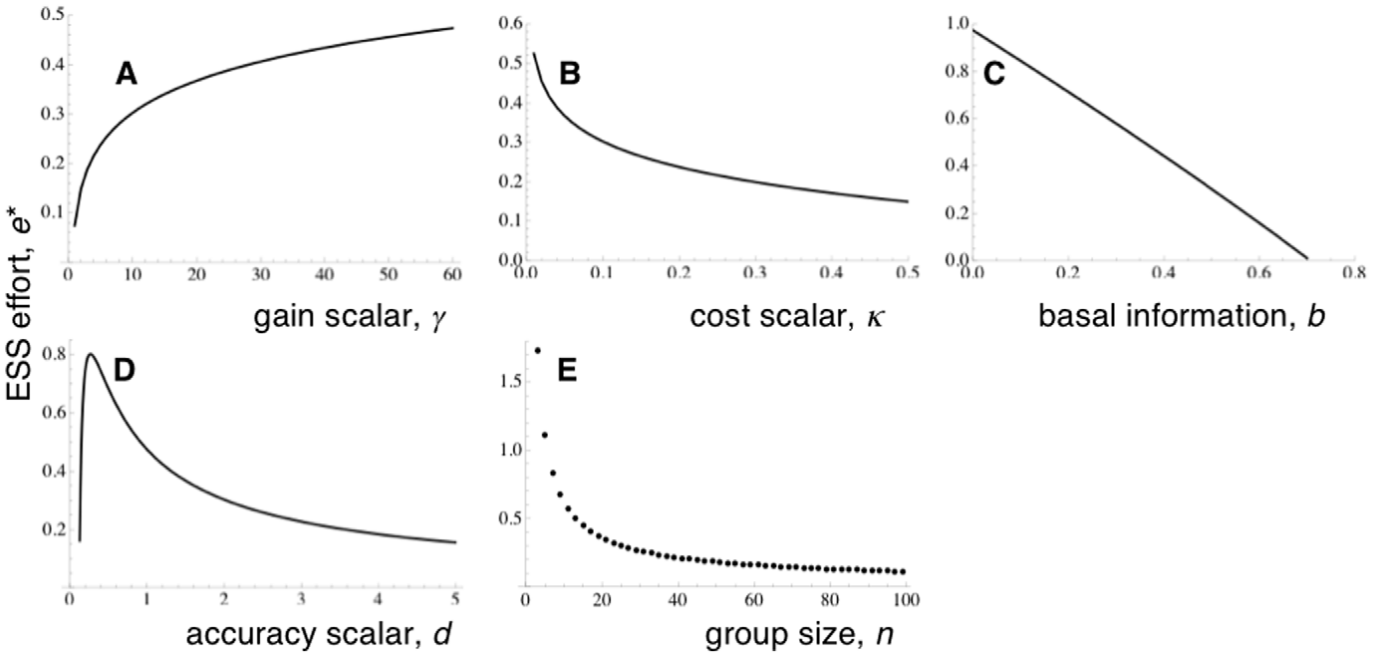
Typical evolutionarily stable levels of effort for the six parameters used in the model. Default values of parameters in the figures: *γ* = 10 units, *b* = 0.5, *d* = 2, *n* = 25 and *κ* = 0.1.

Changing the gain in units of fitness by increasing the scaling constant *γ* leads to an increase in the effort that should be shown by individuals: if there is more to be gained from being correct, it pays to invest more effort (figure 1A). Similarly, if we increase the cost scalar *κ*, the amount of effort should fall (figure 1B).

The size of the basal degree of information correctness, *b*, (where 1−*b* represents the degree of uncertainty about the decision being considered) affects the ESS level of effort: increasing the probability of being correct without having to put in any effort means that the effort made by an individual should fall, until some intermediate value of *b* above which there is no ESS effort (figure 1C) – above this, the costs associated with any effort to increase public information will be to high to make the effort worthwhile. Increasing the accuracy scaling constant *d* leads to an increase in the amount by which any effort decreases the uncertainty in the information, but the effect of increasing this constant on the ESS level of effort to put in varies, where some intermediate value of *d* gives a maximal value of e* (as demonstrated in figure 1D): therefore, increasing *d* could lead to an increase or decrease in e*. Note that figure 1D gives a case where e* is at a maximum at a low intermediate value of *d*. In some parameter sets, no valid solution exists for very small values of *d*. This ties in with the fact that larger values of *b* may not yield a valid solution, as *b* is going to be heavily influential on *I_p_*(*e*) (as described in eqn. 1) when *d* is small.

Group size, *n*, has a distinct effect upon the ESS amount of effort shown within a population. Figure 1E demonstrates that individual effort should decrease as group size increases. Note that once group size has become sufficiently large, the change in ESS effort will be vanishingly small with further increase in group size, and we could therefore suggest that effort is essentially independent of group size in large groups.

## Discussion

The effort an individual puts into improving its information is therefore dependent upon group size, and we would expect to see these changes in effort in any situation where individuals are partially relying upon other group members to process information about the environment. This result is particularly pertinent to studies of vigilance behaviour. Following the theoretical framework proposed by Pulliam [11], standard theory predicts that individual vigilance levels (which I am taking to be akin to effort here) should fall with an increase in group size [12]–[15]. Work related to these predictions usually run on the assumption that scanning by individuals is an independent process, and that no coordination is seen between the members of the group [16]. The model I present here demonstrates, as with the earlier standard models, effort should fall with increasing group size (figure 1E). However, unlike previous models, the model presented here relies upon there being some level of coordination between the individuals. Although there is much work suggesting that vigilance is independent, recently both theoretical [17], [18] and empirical studies of birds [19], [20] and mammals [21]–[25] have suggested that there may be occasions where there is some degree of coordination in vigilance bouts: for example, individuals within multispecies aggregations of gulls display watchfulness that is correlated with the vigilance activity of their neighbours when resting [19]. This means that coordination of activity may happen within vigilant groups, where individuals are monitoring the efforts of others, and responding to them accordingly. Therefore, the framework presented here may give an alternative explanation for empirical observations of vigilance.

This model suggests that as groups become very large, there is relatively little change in the evolutionarily stable amount of effort that an individual should invest. Therefore, for very large groups, ecological factors such as the costs and benefits associated with enhancing personal information will be much more important than the exact size of the group. Note that the model assumes that all individuals have instant access to public information. With larger groups, this is unlikely to be practical, and consideration needs to be given to how individuals could physically assess the intentions of all the group's members. In smaller groups, the exact amount of effort to invest is much more affected by exact group size, but in these groups, it will be much more possible to accurately assess both the size of the group and the intentions of the group's members.

The results I present here require careful testing in a suitable biological system (preferably one where the costs of information gathering can be manipulated): a socially foraging species where the group moves to foraging sites without single individuals taking the lead (such as the plains zebra, *Equus burchellii* [26]), would be a suitable system. Although consideration could be given to cases where all individuals are identical in both their needs and their decision-making processes [27], [28] further consideration needs to be given to cases where some individuals have more influence than others [29]–[34]. Similarly, it is very likely that there will be biological situations where individuals may differ in their inherent qualities [35]–[38], where the group has more than two options to choose between [39], or where there are differences in both information accuracy and knowledge of the group consensus between individuals [35]. Parallel work in the social sciences have explored many different aspects of human social decision-making in public goods games and cooperative problem-solving (*e.g.* [40]–[42]), which could inform our understanding of biological voting games. In particular, including a personal cost of information acquisition in group decision-making processes in addition to the Condorcet majority (*e.g.* [39], [40]) could also give some interesting new insights into the evolution of collective decision-making.

Furthermore, I only consider unrelated individuals in this model: including the effects of relatives benefiting from an individual's actions could well have impacts upon the optimal amount of effort shown, and warrants further investigation. In the current model, individuals can suffer costs from investing effort in improving group accuracy, but will benefit from the group being correct, regardless of whether any other individual in the group is related to them: this could be equated to human competitive systems where group-level actions come from individuals learning the actions of the group [41], rather than from cooperation. I have demonstrated here that living in egalitarian groups may have large effects upon the level of investment that individuals put into group decision-making. This result is particularly relevant to group-living animals that rely on a consensus or a quorum number of individuals to make an accurate decision [6], [36]–[38], [42]–[44].
